# A District Nurse/GP Attachment
*Reprinted from The Medical Officer, Vol. CXVII No. 13, 1967


**Published:** 1967-12

**Authors:** W. B. Fletcher

**Affiliations:** Statistician and Records Officer, City of Bristol Health Department


### A DISTRICT NURSE/GP ATTACHMENT*

### An Evaluation Study

BY

W. B. FLETCHER, A.M.R., F.S.S.

Statistician and Records Officer, City of Bristol Health Department

The district nursing service in Bristol comprises more than 80 nurses working on a geographical basis, of whom 52 have specific case-loads and the others act as reliefs and assistants. Requests for their help are channelled through a central office and then out to the appropriate nurse.

In 1965 consideration was given as to what might be done to enable general practitioners to have closer working relationships with district nurses. A selected number of doctors was asked whether they would welcome the attachment of a nurse, what type of work they thought the nurse would do, and—if she were to work at the surgery—the approximate number of hours she would be required. They were also asked questions on transport and size of the area which was covered by the practice.

The answers were variable, but most thought that secondment of the nurse to the practice would improve personal relationships and enable the general practitioner to confer directly with the nurse about the patient, instead of calling the central unit or leaving notes and telephone messages.

It was thought that the nurse could carry out immunization of children, therapeutic injections, dressings, urine testing, and taking of specimens on behalf of the general practitioner. Preparation of treatment trays, sterilizing instruments, and servicing the doctor's treatment case were also suggested as suitable tasks to be undertaken at the doctor's surgery.

One doctor thought that home visiting by the nurse and doctor might be reduced if a nurse were attending his surgery at regular intervals to give injections, particularly to elderly patients. He thought that a number of geriatric patients could, with a little effort, make their way to the surgery for attention.

All the district nurses were asked at a certain point in time, to examine the cases on their books in terms of the number of GPs involved, and it soon became apparent that most nurses were treating patients from up to 10 or 15 different doctors.

It was decided that one young Queen's nurse would be attached to a small practice of 4,500 patients where two doctors, with secretarial help, were keenly interested to take part in the experiment. In addition to morning surgeries the doctors operated an afternoon appointments system and a limited appointments system at their evening sessions. Their practice was an old established one in a central area, relatively compact, but with a few elderly patients living more than three miles away from their surgery.

In the summer of 1966 the nurse started to work exclusively for the doctors and at that point in time the practice had about ten patients under treatment by the district nursing service.

*Reprinted from The Medical Officer, Vol. CXVII No. 13, 1967

The nurse worked five weekdays with Thursday as her day off. Alternate Saturday afternoons and Sundays were also worked, when she dealt both with patients from the practice and with those of another district nurse who, in turn, coped with the practice patients when the attached nurse was off duty. Holiday absences were covered by other district nurses.

The analyses which follow have been made:— *(a)* of the work at the surgery during the 13 weeks ended mid-January, 1967, and *(b)* of the domiciliary visiting for the second half of 1966.

*(a) Work in the Surgery*

Normally, the nurse attended the surgery on two afternoons each week. The average time per session was 1¼ hours, or rather less than 10 minutes for each patient seen.

The type of work undertaken was principally injections of Jectofer and Cytamen (54 cases) for the older patients, vaccinations against influenza (36 cases) for all ages, and no fewer than 48 measles vaccinations for the under-fives. Other treatments recorded were 10 dressings, 8 vitamin injections, and 4 blood tests, in addition to 16 miscellaneous procedures including ear syringing, antibiotic injections and allergy tests. Only one or two patients seen at the surgery would normally have been visited at home if the special surgery sessions had not been held.

The following is a range of the ages of patients seen at the afternoon sessions during the 13-week period under review:—

| *Age-Groups (Years)* | *Attendances* |
|---|---|
| —15 | 51 |
| 15—44 | 25 (2 males) |
| 46—64 | 52 (27 males) |
| 65+ | 49 (33 males) |
| All ages | 177 |

Having regard to the number of patients at risk the attendances at the nurse's surgery were highest for children under 5 years. The rate per 100 patients per annum for this group was 50, compared with 23 for patients over 65 years. For the remainder of the patients, the attendance rate was less than 10 per 100 patients per annum.

On five days each week the nurse called at the surgery at least once each day to discuss patients' needs and progress with the doctors, and also to collect details of new cases, etc. This work accounted for another 1¾ hours, making a total of 4¼ hours work at the surgery, in addition to time spent visiting the patients in their homes.

*(b) Work on district*

Returns are available of work done month by month for all nurses on the district as well as for the attached nurse.

For all practical purposes it can be assumed that the figures for July to December relate to work performed during the attachment and the nurse's work for this period can be compared with her work during the first half of the year when she covered a fixed area. Unfortunately, however, during her first six months, the nurse did not have the use of a car, but used a bicycle.

The work of the attached nurse can also be compared with the average of the entire nursing staff, equivalent to almost 80 full-time personnel.

During the first half of the year, the single nurse spent 5½ hours a day on the district and averaged 10.2 visits per day or 1.9 per hour. For the entire staff during the same period, the nurses averaged 12 visits per day, and it should be emphasized that almost all the other nurses had motorized transport. Even so, the range was from nine visits per day to 16 per day. On the whole the single nurse worked rather less hours during her period on a district than did the majority of the nurses, although she undoubtedly carried out all that was required of her whilst on the district.

Throughout 1966 the average number of visits per hour and per day and the range of daily visits remained relatively constant for the nurses as a whole. By contrast the work of the attached nurse rose steeply. Her hours of work were increased by a third and the visits per hour by a similar amount. The final result was that her visits per day worked rose by more than 75 per cent.

The following summary shows these differences quite clearly:—

| *Work from July to December,* 1966 (189 *days*) | *Attached Nurse* | *Average for all other full-time Nurses* |
|---|---|---|
| Hours (surgery 12%, district 88%) ... | 905 | 716 |
| Domiciliary visits ... ... ... | 2,222 | 1,464 |
| Days worked ... ... ... ... | 122 | 125 |
| Average hours per day worked ... | 7.4 | 5.7 |
| Average visits per hour worked ... | 2.5* | 2.0 |
| Average visits per day worked ... | 18.2 | 11.8 |

*Based on total hours worked. If based only on hours on district, the figure becomes 2.8 per hour.

*Mileage*

The mileage of all the district nurses is not accurately known for the period under review as many are on a fixed car allowance after a period of recording, but it is known that a mileage of 240 to 300 per month is fairly common. For the attached nurse the mileage was at least double the average figure of nurses working in confined districts. In terms of cost this attachment probably adds about £100 per annum to her travelling costs. Whether this difference will be maintained over a full year is difficult to say, as during the later months there appeared a downward trend, although the number of home visits remained fairly constant.

### DISCUSSION

To use a full-time nurse to look after 4,500 patients may appear to be a luxury when one considers that there are about 5,500 people per full-time nurse in the city. We also must take into account a certain amount of relief given to the attached nurse during holidays and normal off duty. We know from our annual statistics that two-thirds of the work of district nurses is for people over the age of 65. It is estimated that there are about 60,000 people aged 65 or over in the city, or some 750 old persons per full-time district nurse. The actual number in the practice is 768.

Although the nurse started her attachment with about 10 domiciliary cases, the number has steadily risen and is now in the region of 40. This figure is certainly as great as the average for all the other 52 nurses with specific case loads. Some of the other nurses with large case loads obtain help from the relief nurses who do not cover a specific district.

An analysis of case-loads current at the end of 1966 showed that the 52 nurses with defined districts averaged 40 cases each, of whom 28 were over the age of 65 years. A similar examination of the cases held by the attached nurse revealed she had 39 patients in her care, of whom 33 were over 65 years.

Whatever the average case-load, however, it has been shown that the attached nurse has worked longer hours and seen more patients in their homes than the average nurse, as well as spending an eighth of her time at the doctors' surgery. The latter aspect has proved of very real benefit both to the patients and the doctors alike. Since her attachment to the practice, the doctors have called upon the district nurse more frequently than when they were required to contact a central office and give information over the 'phone for onward transmission. Before the attachment they were also under the impression that there was a shortage of district nurses and they often curtailed their requests. This belief was found to be held by many doctors in the city and is supported by the findings of Hockey (1966).

To be able to speak directly to the nurse and for her also to report back directly is an undoubted advantage to everyone. Discussion of cases enables a free flow of information to the advantage of doctor, nurse, and, most important of all, the patient. Duplication of instructions is avoided and the little notes on the mantelpiece—either requesting or (less frequently) thanking —have disappeared. The doctors also now prescribe iron injections more often in the place of oral iron, which means quicker relief to the anaemic patient. A further benefit to the patient is that more dressings are given by the nurse, which frequently means faster healing than if the task were undertaken by the patient.

Patients with chronic conditions who suffer from inter-current illnesses are quickly noticed by the nurse, and this results in a visit from the general practitioner. One such case concerned an old lady with early pneumonia which the doctor felt might well have proved fatal had it been left to a relative or the patient to call him.

Some reduction of visiting by the general practitioners has been possible in cases of pneumonia. Where the nurse is visiting daily to give antibiotics, the doctor can rely upon her to report back to him any untoward conditions which require his attention.

The doctors express unqualified satisfaction with the arrangement, but the nurse herself goes even further. In spite of the extra work, she indicates that her satisfaction with the job has increased beyond her most optimistic expectations. She had been thinking of giving up district nursing before the scheme came into being, but all such thoughts have now been dispelled. The nurse now identifies herself with the practice and her sense of responsibility matches that of the doctors themselves; this is reflected in her making odd calls on her day off to patients in particular need. There can be little doubt that the patients feel they are being treated by a team, each member knowing what is going on.

What then are the disadvantages? There was early concern at the increased transport cost. Undoubtedly, if the doctors limited the area in which they practise, they would reduce not only their own travelling time, but also that of any attached ancillary staff. This position, however, will not be achieved for many years although some of the more enlightened doctors are moving to this end.

Mileage economy for the nurse has, to some extent, been achieved by the doctors having an orderly method of passing cases to her each morning, so that she may plan her visiting in the knowledge that she is unlikely to have odd messages later telephoned through to her. In fact, the doctors exercise the same care and consideration in calling the nurse to visit as they urge their own patients to use in calling them. It is likely that a journey of one mile will not take twice as long as one of half a mile. The nurse can also be advised by the doctor of any peculiarities of where the patient lives—possibly the best way to get there—and other time-saving bits of information.

There still remains the cost of car allowances. If one takes into account the salary of a district nurse, her state insurance, pension fund, uniform and travelling, etc., the total sum is in the region of £1,350 per annum, of which travelling costs average about 10 per cent.

It has been shown that a full-time nurse working in a district averages about 2,900 visits per annum, and that the attached nurse was making visits at the rate of 4,000 per annum for the second half of 1966. In terms of cost per visit, the following figures show how this compares:—

| | *Total Visits* | *Salary, Uniform, etc.* | | *Travelling* | | *Total* | |
|---|---|---|---|---|---|---|---|
| | | *Per Annum* | *Per Visit* | *Per Annum* | *Per Visit* | *Per Annum* | *Per Visit* |
| | | £ | s. d. | £ | s. d. | £ | s. d. |
| District nurse average | 2,900 | 1,215 | 8 4 | 135 | 0 10 | 1,350 | 9 2 |
| Attached nurse | 4,000 | 1,215 | 6 1 | 250 | 1 3 | 1,465 | 7 4 |

To attach all nurses to general practitioners may well increase the visits requested, as indeed has occurred in the present experiment. No frivolous calls have been made upon the nurse's time, so there would seem to be a relatively large number of nursing requirements which the existing system is not being asked to meet.

The difficulties of supplying relief for sickness, holidays and time off are increased where a nurse works alone. To extend attachment of nurses would be simpler where doctors are working in larger groups, particularly at health centres, and further study is being given to this aspect.

One point should be borne in mind. To budget for an even load of cases in a given geographical area is undoubtedly a difficult problem. For the city as a whole in any one year, one-third of all cases are on the books for a week or less; two-thirds are discharged within a month and only about 10 per cent stay on the books for a year or more.

There is obviously a degree of judgment which a general practitioner can use in deciding whether it is reasonable to call in a nurse. This must depend upon the resources available and it would seem that when the general practitioner can see his nurse is at full stretch he can go a long way to cut down additional work. Equally, when it comes to a nurse being short of patients, the doctor may off-load some less essential matters on to her. This prevents boredom and, above all, shows the nurse that she is getting some personal consideration from one who is in close touch with her work. There is no doubt that the nurse taking part in this experiment has found the work very much more rewarding than under the old scheme.

### SUMMARY

The attachment of a district nurse to a practice of 4,500 patients in a city is discussed.

The main advantage of the scheme—apart from greater job satisfaction—has been better communicaions for the doctor and nurse and a much greater increase of visits per hour employed, as well as more effective hours being worked by the nurse.

Miles travelled by the nurse were double the average for the unattached nurses, but represent an approximate increase of less than 10 per cent in the total cost of employing the nurse.

The attachment of a nurse or nurses to larger groups of doctors, particularly those working in health centres, should be seriously considered.

### ACKNOWLEDGEMENTS

I would like to thank Professor R. C. Wofinden, MOH, City of Bristol, for his help and encouragement in the writing of this paper, and Dr. R. M. Brown and Dr. R. P. Golding for making available information about their practice. I am grateful to Miss Howe, who has enthusiastically recorded many additional items of information, and to Miss G. M. Grazier and her entire staff of district nurses who have provided the general statistics on which this paper is based.

### REFERENCE

Hockey, Lisbeth (1966): " Feeling the Pulse—A Study of District Nursing in Six Areas ". Queen's Institute of District Nursing, London.

